# Screening for Monkeypox Infection in Asymptomatic High-Risk-Behaviour Men Having Sex with Men (MSM)

**DOI:** 10.3390/idr14050081

**Published:** 2022-10-17

**Authors:** Julia Pestel, Hanna Matthews, Stefan Schmiedel, Anja Hüfner, Sabine Jordan, Robin Luca Scheiter, Marc Lütgehetmann, Dominik Nörz, Olaf Degen

**Affiliations:** 1 Infectious Disease Clinic, University Medical Center Hamburg-Eppendorf (UKE), Martinistraße 52, 20246 Hamburg, Germany; 2 I. Department of Medicine, University Medical Center Hamburg-Eppendorf (UKE), 20246 Hamburg, Germany; 3 Institute of Medical Microbiology, Virology and Hygiene, University Medical Center Hamburg-Eppendorf (UKE), 20246 Hamburg, Germany

**Keywords:** monkeypox, screening, HIV, STD

## Abstract

**Background:** Since the outbreak of monkeypox in formerly non-endemic countries, we have included a screening for monkeypox in our sexually transmitted diseases (STD) routine in patients with high-risk behavior, as it is mainly transmitted through close skin to mucous membrane contact with infected individuals. **Methods:** Between 16 June 2022 and 14 July 2022, we screened 53 MSM with high-risk behavior for monkeypox acquisition in an observational prospective cohort trial. We complemented the throat and anal swabs for chlamydia and gonococci with monkeypox using polymerase chain reaction (PCR). In addition, all patients participated in a questionnaire survey about their risk behavior and previous STD in their medical history. **Results:** None of the 53 participants had tested positive for the monkeypox virus. One patient was diagnosed with syphilis and one with an oral and anorectal chlamydia infection. **Conclusions:** STD screening in high-risk populations is a valuable tool to detect asymptomatic patients for chlamydia, gonococci, HIV, hepatitis B and C and syphilis. Based on our small cohort, monkeypox screening in asymptomatic MSM patients in areas of low prevalence does not seem to be an appropriate approach to deal with the ongoing outbreak. Therefore, we recommend to focus more on vaccinations, targeted nonstigmatizing information and behavior recommendation for risk populations, and to engage further investigations.

## 1. Introduction

Monkeypox is a viral zoonotic infection that was first reported as an outbreak in non-endemic countries in May 2022 with exclusively human-to-human transmission [1]. Since May 2022, 3188 cases have been reported in Germany as of 16 August 2022 [2]. Most cases have been identified in men who have sex with men (MSM) after close skin to mucous membrane contact of active lesions during sexual intercourse. Prodromal symptoms are systemic illness with fevers, myalgia and lymphadenopathy. Later on, patients develop characteristic lesions especially in the genital or oral region. In some cases, lesions appear without initial prodrome [3].

In our Infectious Disease Clinic at the University Medical Center Hamburg-Eppendorf, Germany, we see patients taking pre-exposition prophylaxis (PrEP) or postexposure prophylaxis (PEP) to prevent getting HIV, or people living with HIV, for a sexually transmitted diseases (STD) check. As monkeypox seems to be transmitted mainly by sexual contact [4], we included monkeypox screening in our regular STD check using anorectal and oropharyngeal swabs.

## 2. Materials and Methods

In an observational prospective cohort, we screened for monkeypox in oropharyngeal and anal swabs from 53 consecutive patients between 16 June 2022 and 14 July 2022. We asked all individuals with changing sexual partners (PrEP-, PEP users and people living with HIV) to participate additionally in a detailed online REDCap (research electronic data capture) questionnaire. In this survey, we collected data about sexual orientation, sexual risk behavior in the last 6 months and sexually transmitted diseases in their medical history. All subjects gave their informed consent for inclusion before they participated in the study. The study was conducted in accordance with the Declaration of Helsinki. The protocol was approved by the Ethics Committee of the Ärztekammer Hamburg (Project identification code: PV7127, date of approval 4 February 2020).

Our regular STD check includes serologies for syphilis, HIV and hepatitis C and first-void urine and swabs (anorectal and oropharyngeal) for chlamydia and gonococci. These swabs were also used for the screening of monkeypox. All samples were aliquoted and inactivated by adding Roche polymerase chain reaction (PCR) media (Roche Diagnostics, Switzerland) prior to molecular diagnostics. In order to detect monkeypox virus-DNA, we applied a quantitative real-time qPCR on a cobas5800 or cobas6800 system [5].

For descriptive analysis, Microsoft Excel (Version 16.0.5332) was used.

## 3. Results

A total of 53 MSM asked to participate in this screening (for detailed characterization see Table 1). All identified themselves as men who have sex with men (MSM). The median age of the study population was 35 years old (range 19 to 52 years). A total of 34 (64%) out of the 53 patients answered in our survey that they had had sex with five or more men in the past 6 months (range 1 to >9 sexual partners, median 4). A total of 2 (3.77%) patients did not answer this question. A total of 30 participants (57%) had had a STD in their medical history (range 0 to 6, median 3). A total of 40 (75.5%) patients were using PrEP, 6 (11.3%) patients were living with HIV, 5 (9.4%) MSM patients did not use PrEP and 2 (3.8%) patients took PEP to prevent getting HIV. A total of 4 (7.5%) anal swabs were not taken due to no passive anal intercourse.

None of the patients showed a positive result for monkeypox. In our routine STD check, we detected and treated two (3.8%) sexual transmitted diseases. One patient was diagnosed with an oral and anal chlamydia infection and one patient had a positive serology test for syphilis. No patient tested positive for HIV or hepatitis C.

## 4. Discussion

Based on CDC recommendations [6], we know that screening patients with high-risk profiles is a powerful tool to detect sexually transmitted diseases. In this prospective study, the majority of the screened participants were PrEP or PEP users with a high number of sexual partners in the last 6 months. We perceive the study group as representative for the MSM population in Northern Germany, even though the incidence of STD was rather low, with 3.7% (2/53) in comparison to 20% in other study groups in Germany [7].

Since the outbreak of monkeypox in formerly non-endemic countries, we added a test for monkeypox in our STD routine. Human monkeypox is a zoonotic orthopoxvirus with presentation similar to smallpox. Sarkar et al. showed already in 1973 that smallpox can be detected in the upper respiratory tract of asymptomatic contacts [8]. During endemic monkeypox outbreaks in the previous years, asymptomatic cases were not investigated.

In this trial, none of the patients had an asymptomatic monkeypox infection. In comparison to our results, De Beatselier et al. found three asymptomatic patients with a PCR-positive anal swab for monkeypox in stored samples from 224 MSM in a retrospective trial in Belgium in June 2022 [9]. The results of De Beatselier et al. might lead to the conclusion that asymptomatic carriers could play a key role in virus transmission. Unfortunately, De Beatselier et al. could not confirm the monkeypox positivity of the asymptomatic cases in a second sample from different body parts or at a different time. As these findings come from a retrospective analysis, we cannot retrace if the included patients really were asymptomatic since there was no clinical examination. A recent prospective study from Paris, France, found 13 (6.5%) anorectal swabs being positive for the monkeypox virus in 200 asymptomatic men [10]. Only two of them developed symptoms in the follow-up. Whether the positive PCR results indicate viral replication and possible transmission is unknown. Further investigations are needed to address these uncertainties.

The significance of our analysis is limited since we evaluated only a small number of patients. In Hamburg, over 152 monkeypox cases have been documented until 15 August 2022, with the 4 weeks of our screening period showing the highest number of reported cases so far [11]. In Berlin, as an international hotspot with 1512 reported cases (16 August 2022) out of over 38,000 worldwide [12] the incidence is about 5 times higher. In our collective, none of the screened participants reported contact with a confirmed monkeypox case in the initial questioning.

Especially in international communities such as Berlin, awareness campaigns in healthcare settings should focus on reliable information about signs and symptoms as well as behavior recommendations for risk populations. Providing targeted nonstigmatizing information to reduce the risk of transmission and identify, screen and examine contact persons could be an appropriate approach to treat the ongoing outbreak. In addition, based on the recommendation of the Standing Committee on Vaccination (STIKO) in Germany from 21 June 2022, targeted vaccination programs with the pox vaccine Imvanex/Jynneos as a postexposure prophylaxis (PEP), pre-exposure prophylaxis (PrEP) for persons with increased risk (MSM, frequent change of sexual partners) and personnel in specialized laboratories handling with infectious samples [13] should be established.

Based on our small cohort, monkeypox screening in asymptomatic MSM patients in areas of low prevalence does not seem to be a suitable tool to detect asymptomatic infections. Nevertheless, in high-prevalence settings this might still be reasonable to prevent further infections via asymptomatic individuals.

## Figures and Tables

**Table 1:** Sexual and demographic characterization.

	Numbers of Sexual Partners in the Past 6 Months										
	1	2	3	4	5	6	7	8	9	>9	Unknown
Numbers of patients (n = 53)	6	2	5	4	4	2	4	3	-	21	2
**Number of STD in medical history**											
None (n = 23)	4	2	3	1	-	2	1	2	-	7	1
1 (n = 13)	1	-	1	1	3	-	1	-	-	5	1
2 (n = 7)	1	-	1	-	-	-	2	-	-	3	-
3 (n = 2)	-	-	-	1	-	-	-	-	-	1	-
4 (n = 3)	-	-	-	1	-	-	-	1	-	1	-
5 (n = 3)	-	-	-	-	-	-	-	-	-	3	-
6 (n = 2)	-	-	-	-	1	-	-	-	-	1	-
**Risk group (n = 53)**											
PrEP (n = 40)	5	1	4	2	3	2	3	3	-	16	1
People living with HIV (n = 6)	-	-	-	2	1	-	-	-	-	2	1
MSM without PrEP (n = 5)	1	-	1	-	-	-	1	-	-	2	-
PEP (n = 2)	-	1	-	-	-	-	-	-	-	1	-

## Data Availability

The data presented in this study are openly available.
